# Flushing: A Diagnostic Dilemma

**DOI:** 10.7759/cureus.15515

**Published:** 2021-06-08

**Authors:** Rekha Ravikumar, Salil Avasthi, Deepti Avasthi

**Affiliations:** 1 Internal Medicine, Mercy Health - St. Vincent Medical Center, Toledo, USA; 2 Pulmonary Critical Care, Mercy Health - St. Vincent Medical Center, Toledo, USA

**Keywords:** carcinoid syndrome, neuroendocrine tumor, menopause, flushing, hot flashes, delayed diagnosis

## Abstract

Carcinoid tumors are uncommon tumors that are often diagnosed in later stages of the disease due to their indolent nature, vague clinical presentation and overlap of symptoms with other conditions. We report a case of rectosigmoid carcinoid tumor which was found incidentally on screening colonoscopy in an elderly woman that went undiagnosed for several years due to confounding effects of symptoms of post-hysterectomy menopause with that of carcinoid syndrome. Persistent episodic flushing with or without diarrhea not resolved with standard treatment should lead to suspicion of neuroendocrine tumors (NETs). This case report emphasizes the need to broaden our perspective of menopausal symptoms and pay attention to the characteristic clinical symptoms of NETs.

## Introduction

Neuroendocrine tumors (NETs) secrete biologically active substances which include serotonin, tachykinin, histamine and prostaglandin. These biologically active mediators when released into circulation result in a constellation of symptoms such as episodic flushing, diarrhea, wheezing, tachycardia and blood pressure fluctuations. Together these symptoms are known as carcinoid syndrome. Carcinoid syndrome is found in fewer than 10% of NETs. According to the Surveillance, Epidemiology, and End Results database, the incidence of NET is about 5 per 100,000 people [1,2]. The incidence of NET has increased in the United States and around the world over the years. This trend in increased incidence is partly attributed to incidental discovery during endoscopic screening programs [3]. A review of the literature has shown that the average time from symptom onset to diagnosis is more than nine years [3]. The presence of atypical symptoms and an overlap of symptoms with other diseases are the most common reasons behind the delay in diagnosis [4]. The aim of this case report is to emphasize the need to broaden our differential diagnoses for persistent menopausal symptoms, stratify patients who need further testing to rule out endocrine causes and establish a guideline for diagnosis of menopause.

## Case presentation

A 62-year-old female presented to the Internal Medicine clinic to establish care. She had no chief complaints. Her review of systems revealed presence of chronic flushing, hot flashes, and mild intermittent diarrhea for 10 years. She denied abdominal pain, back pain, palpitations, shortness of breath or wheezing. Patient smoked half a pack of cigarettes a day and consumed alcohol and marijuana occasionally. Family history was significant for ovarian cancer in her mother.

Medication history revealed the use of oral conjugated estrogen 0.3 mg for nine years for her chronic hot flashes. Her Obstetrician discontinued the treatment one year ago on account of only partial relief with the medication and high risk of complications from long-term use of estrogen. Her hot flashes worsened with discontinuation of oral estrogen.

Her past medical history was significant for a hysterectomy at the age of 25 years and rectosigmoid polypectomy at the age of 56 years. She had four sub-centimeter rectosigmoid polyps incidentally found on a screening colonoscopy (5/20/2014) for colon cancer at that time. Pathology results showed the presence of hyperplastic polyp in three of the polyps and a grade 1 well-differentiated low-grade NET in one of the polyps. Both the stalk and the margins of the resected polyp were positive for NET. No imaging was done at this time. Patient was requested to get surveillance colonoscopy in one year. Patient being ignorant of her diagnosis did not seek any medical help for surveillance of her tumor and polyp.

Blood work done on 8/4/2020 showed elevated chromogranin A of 276 ng/ml (normal 0-160 ng/ml), elevated serotonin of 230 ng/ml (normal 50-220 ng/ml) and urine 5 HIAA of 6 mg/l (patient had improper collection of 24-hour urine; normal <15 mg/l). CT scan of abdomen and pelvis with contrast was unremarkable for liver metastasis.

**Figure 1:**
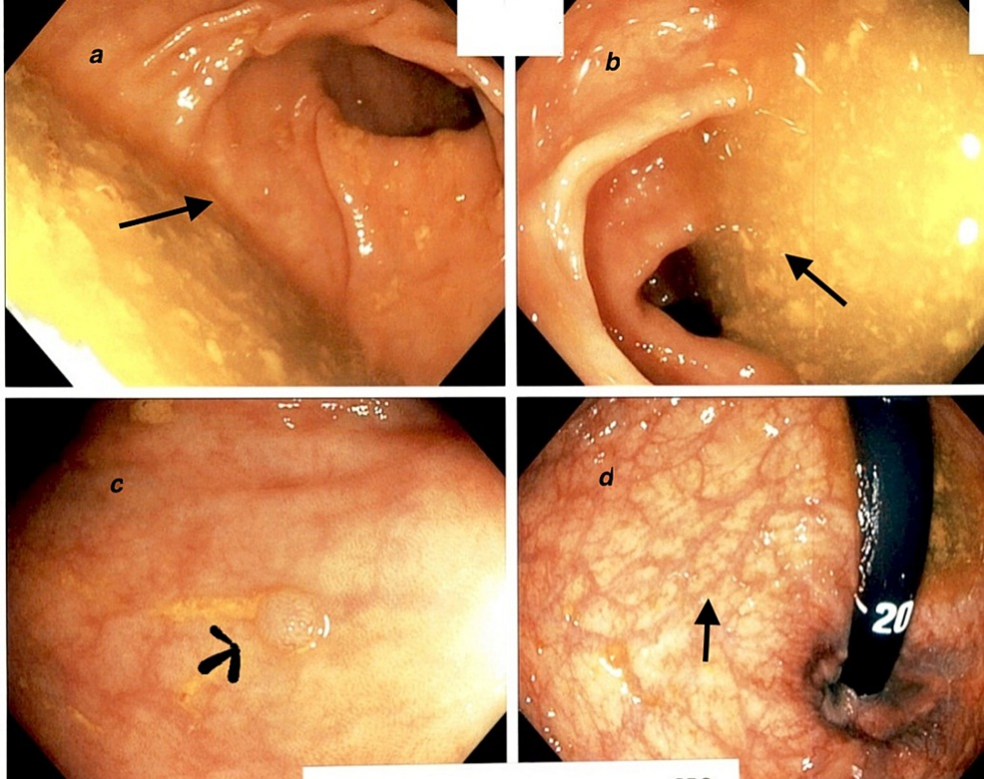
(a) and (b) are rectum. (c) polypoid mass in rectum. (d) sigmoid colon

**Figure 2:**
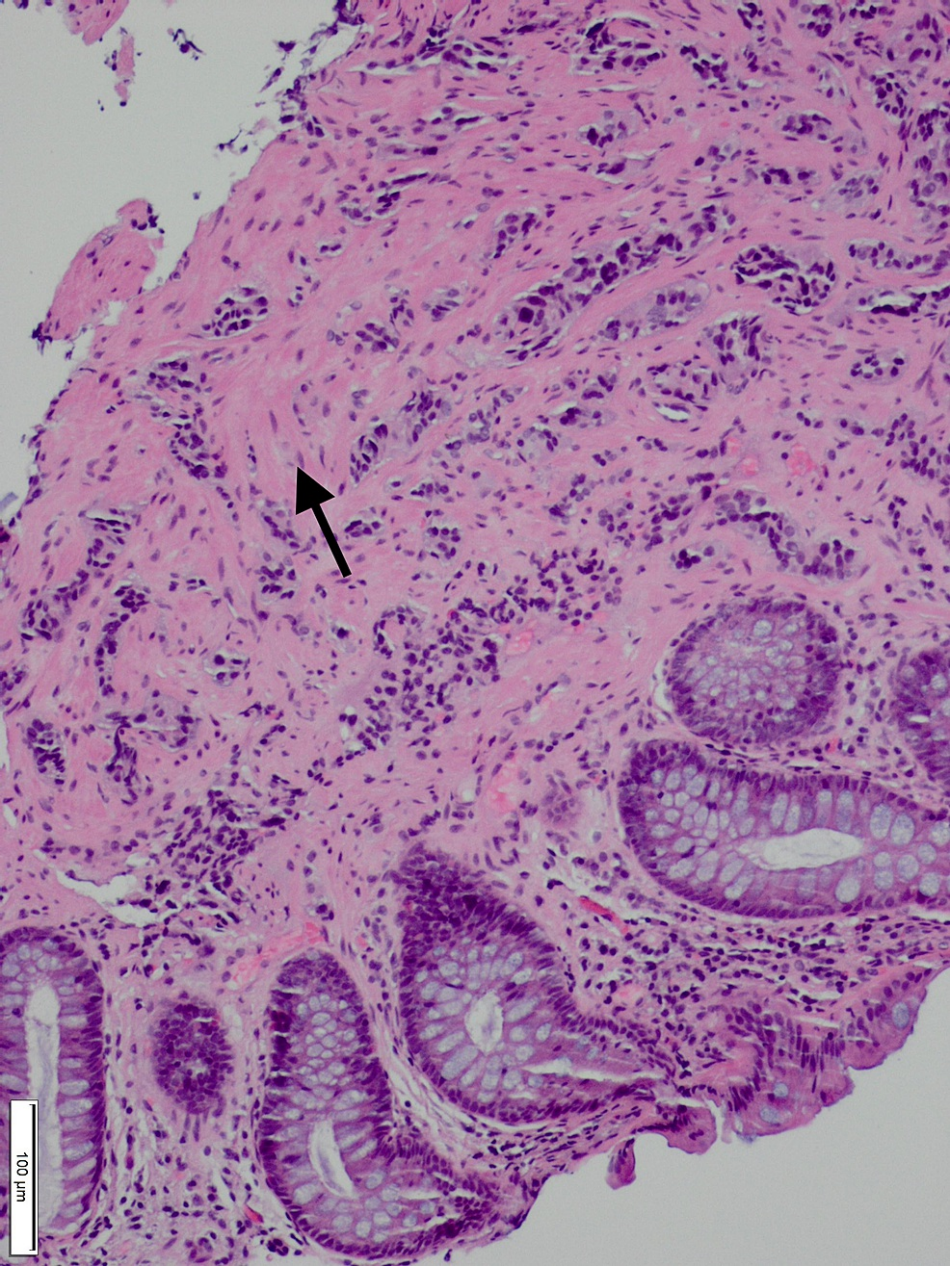
Normal mucosal glands are present on the right and bottom of the slide with nests of well differentiated neuroendocrine tumor (carcinoid tumor, small blue cell groups) in the submucosa and superficial muscularis propria in the left and upper portion of the slide. (Hematoxylin and Eosin, 100X)

**Figure 3:**
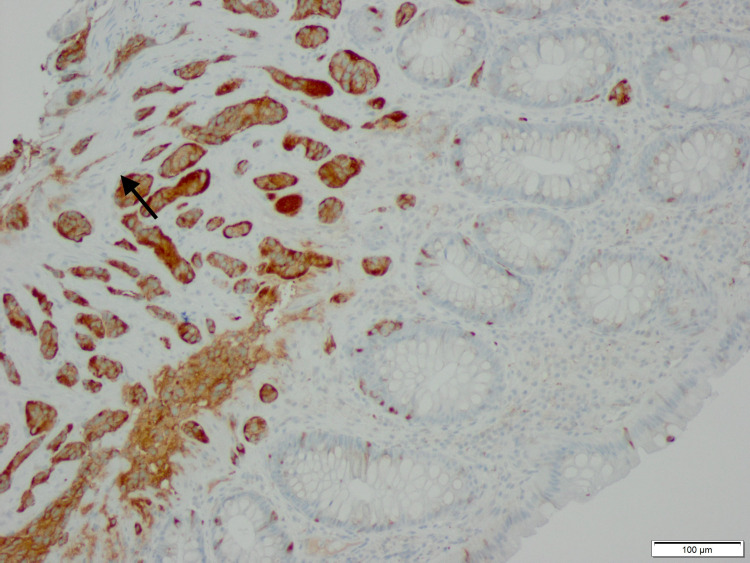
Corresponding synaptophysin immunostain demonstrates brown staining in isolated normal neuroendocrine cells in mucosal crypts in the right and bottom of the slide and highlights tumor nests in the submucosa and muscularis propria in the left and upper portion of the slide. (100X)

The repeat colonoscopy done on 12/17/2020 revealed presence of a 4 mm polypoid mass (Figure 1) with a yellow surface in the rectum and 4 mm tubular adenoma in ascending colon and sigmoid colon. Later the pathology findings (Figures 2, 3) indicated recurrence of a Grade 1 well-differentiated NET/carcinoid in the rectum. Immuno-stains for chromogranin A and synaptophysin were positive in the infiltrating tumor nests in the lamina propria, confirming the neuroendocrine tumor. Upper gastrointestinal endoscopy was done for evaluation of iron deficiency anemia, revealed mild gastritis and was negative for tumors. Patient is scheduled for rectal endoscopic ultrasound (EUS) for the staging of the primary rectal tumor following which appropriate treatment will be initiated. Patient is following with oncologist and will be referred to a surgeon as she is a surgical candidate due to recurrent disease.

## Discussion

Our patient’s clinical presentation of flushing is one of the most commonly encountered medical problems in Internal Medicine and Family Medicine outpatient clinics. Most often these female patients are diagnosed as perimenopausal based on their age and symptoms. Often laboratory evaluation is not ordered to establish the diagnosis. We acknowledge that menopause is the most common cause of flushing in women and that avoiding the confirmatory hormonal test reduces the cost burden on the healthcare system. But one cannot ignore the fact that this approach raises the incidence of diagnosis bias and treatment errors by physicians and health care providers. A more careful study of the symptoms, proper staging of the disease and the disease patterns of NET and menopause may help evaluate the vasomotor symptoms better and develop a pragmatic approach to the treatment.

Vasomotor symptoms are generally described as night sweats, hot flashes and flushing. The cause for flushing is thought to be multifactorial and has been attributed to substances like prostaglandin, substance P, kinins and histamine [5]. Flushing may be autonomic-mediated or vasodilator-mediated [6]. Autonomic-mediated flushing includes fever, menopause, emotional blushing and those caused by neurologic disorders. Some causes of vasodilator-mediated flushing include carcinoid syndrome, rosacea, medications, alcohol, pheochromocytoma and serotonin syndrome. Menopausal flushing is mediated by hypothalamic thermoregulatory dysfunction induced by estrogen withdrawal, while on the contrary in carcinoid, flushing happens from cutaneous vasodilation of superficial blood vessels due to the action of circulating vasodilator substances. Flushing is the most frequent symptom of carcinoid syndrome and occurs in 85% of cases. It presents as faint pink to red discoloration of the face and upper trunk and is provoked by tyramine-containing foods such as blue cheese or red wine. Each episode usually lasts one to five minutes but may persist for several hours during the later stages of the disease. Other symptoms of carcinoid syndrome may include diarrhea and wheezing. The incidence of the triad of these symptoms is low and occurs in less than 30% of the cases. Diarrhea is of a secretory type and mostly occurs during fasting. Unlike bronchospasms and wheezing, diarrhea is usually not preceded by flushing.

The type of flushing in carcinoid syndrome also varies depending on the site of the origin of the carcinoid tumor. Tumors arising from the embryological foregut (stomach, lung, pancreas, ovaries and biliary tract) produce characteristic bright salmon pink flushing and are associated with telangiectasias and hypertrophy of neck and facial skin whereas tumors arising from the midgut (distal duodenum to the colon) produce pink or reddish flushing involving the face and upper trunk to the nipple line [2-5].

NETs located in the gastrointestinal (GI) tract release serotonin, which is degraded by the liver, and symptoms do not occur unless the tumor has metastasized to the liver. The incidence of carcinoid tumors is 1.5 per 100,000 population [2]; only 10% of this population in whom liver metastasis has occurred develop carcinoid syndrome. Lungs and liver metabolize the biologically active substances released by NET and hence symptoms do not occur until metastasis ensues. However large primary tumors of the gut with extensive nodal metastasis without liver metastasis can also result in carcinoid syndrome. In addition, bronchial, gastric, appendiceal and rectal carcinoid tumors can occasionally produce carcinoid syndrome even in the absence of metastasis because of their access to the systemic circulation. The presentation with metastatic disease accounts for 12-22%.

Almost 80% of menopausal women experience hot flashes, and only 20-30% seek medical help [7]. Menopausal hot flashes start as a sensation of heat in the upper chest and face that spreads to the rest of the body. Each episode lasts for two to four minutes and is associated with sweating followed by chills and restlessness. Women over the age of 40 years who present with sleep pattern changes, hot flashes and irregular menstrual cycle are considered to be premenopausal. Menopause is diagnosed in a healthy woman over age 45 years, when amenorrhea lasts for 12 months in the absence of other biologic and physiologic causes. It has been established that there is a limited role for serum follicle-stimulating hormone (FSH) in the diagnosis of menopause in women >45 years of age [8]. But menopause in women who have undergone hysterectomy or endometrial ablation cannot be determined using menstrual bleeding criteria. Therefore, supportive criteria, including assessment of menopausal symptoms and biochemical data, are needed. A similar diagnostic dilemma can occur in women taking oral contraceptive pills or women with underlying menstrual disorders like polycystic ovarian syndrome. In these settings, we suggest the measurement of FSH concentrations. A serum FSH >25 international units/L, particularly in the setting of hot flashes, is suggestive of the late menopausal transition [9] and a higher value of FSH (in the 70 to 100 international units/L range) confirms the menopause [10].

Menopause symptoms last an average of 4.5 years, following a person's last period, and 7.4 years in total, according to a study published in 2015. It is vital to look into other causes of vasomotor symptoms in menopausal women especially when it is unresolved for eight years and more than 10 years in African Americans [11].

We suggest additional endocrine testing should include liver function test (LFT), prolactin, thyroid stimulating hormone, serum catecholamines, serum metanephrines, chromogranin A (CgA) levels and serum tryptase to rule out pathologic causes of vasomotor symptoms in patients with unresolved, prolonged or worsening symptoms. A 24-hour urinary 5-HIAA is a widely accepted screening test for carcinoid syndrome. It is to be noted that several substances that can affect the urinary 5-HIAA and result in false positive tests include fruits such as avocados, pineapples, bananas, and plums and drugs like nicotine, acetaminophen, caffeine, ephedrine, phenobarbital, phentolamine and melphalan.

Abdominal CT scan is the imaging of choice for tumor staging. However, CT scan cannot detect small tumors of the jejunum, ileum and appendix. In such cases, CT enterorrhaphy has been shown to be sensitive. Triphasic CT of the liver can be considered in cases of liver metastasis. Somatostatin-based imaging is used for scintigraphy. CT chest is used for bronchial carcinoid tumors and echocardiography for carcinoid heart disease.

Management of NETs is multidisciplinary ranging from medical management of symptoms to surgical removal of the lesion. For small stable lesions, observation is recommended. Somatostatin analogues (SSA) are the treatment of choice for metastatic, progressive or well-differentiated carcinoid tumors. A tryptophan hydroxylase inhibitor, trilostristat ethyl, has been approved as adjunctive therapy to SSA. Antihistamines are useful for the symptomatic treatment of flushing.

Surgical debulking, which can help reduce symptom burden or mass effect, is indicated for metastatic NET. Patients should be monitored clinically and by measuring natriuretic peptides for development of carcinoid heart disease

The median survival rate of well-differentiated NET with local, regional or metastatic disease is 223, 111 and 33 months respectively. Localized tumors arising from the appendix and rectum have a good prognosis with a five-year survival rate exceeding 90%, whereas the median survival rate of poorly differentiated NET is only 10 months [12].

## Conclusions

Various conditions cause flushing and the diagnosis of the underlying etiology can be challenging especially in women of the menopausal age group. A screening evaluation to rule out an underlying NET can be considered in women with persistent symptoms for more than eight years. Measurement of urinary 5HIAA and a serum FSH is a validated and cost-effective method to evaluate the flushing; however, there are no current guidelines for their mass application in the community setting. Further studies are needed to understand the cost and benefit of these screening tests to improve the economic burden of this disease.
